# Fully Recyclable Bio-Based Epoxy Formulations Using Epoxidized Precursors from Waste Flour: Thermal and Mechanical Characterization

**DOI:** 10.3390/polym13162768

**Published:** 2021-08-18

**Authors:** Francesca Ferrari, Carola Esposito Corcione, Raffaella Striani, Lorena Saitta, Gianluca Cicala, Antonio Greco

**Affiliations:** 1 Department of Engineering for Innovation, University of Salento, Via Arnesano, 73100 Lecce, Italy; francesca.ferrari@unisalento.it (F.F.); raffaella.striani@unisalento.it (R.S.); antonio.greco@unisalento.it (A.G.); 2 Department of Civil Engineering and Architecture (DICAR), University of Catania, Viale Andrea Doria 6, 95125 Catania, Italy; lorena.saitta@phd.unict.it (L.S.); gianluca.cicala@unict.it (G.C.)

**Keywords:** epoxidation, thermoset recycling, organic waste

## Abstract

Organic wastes represent an increasing pollution problem due to the exponential growth of their presence in the waste stream. Among these, waste flour cannot be easily reused by transforming it into high-value-added products. Another major problem is represented by epoxy-based thermosets, which have wide use but also poor recyclability. The object of the present paper is, therefore, to analyze both of these problems and come up with innovative solutions. Indeed, we propose a completely new approach, aimed at reusing the organic waste flour, by converting it into high-value epoxy-based thermosets that could be fully recycled into a reusable plastic matrix when added to the waste epoxy-based thermosets. Throughout the research activity, the organic waste was transformed into an epoxidized prepolymer, which was then mixed with a bio-based monomer cured with a cleavable ammine. The latter reactant was based on Recyclamine™ by Connora Technologies, and in this paper, we demonstrate that this original approach could work with the synthetized epoxy prepolymers derived from the waste flour. The cured epoxies were fully characterized in terms of their thermal, rheological, and flexural properties. The results obtained showed optimal recyclability of the new resin developed.

## 1. Introduction

The use of epoxy-based composites is widely accepted in different fields. In the aerospace sector, epoxy resins are used because of its low cost and suitability for producing large structures. Recent studies reported novel technologies for producing enhanced composites for the aeronautical field. Zotti et al. [1] developed PDA-coated silica nanoparticles as filler for a common aeronautical epoxy resin, improving the mechanical properties, the damage resistance, and the thermal stability with respect to the neat matrix. Liquid resin infusion of epoxy resins is well established in the transportation and naval sectors. In the civil sector, the use of epoxy composites is widely accepted for semistructural and structural applications. An increasing interest was recently devoted to the epoxy–timber composites in the construction field. Awad et al. [2] studied the effect of calcium sulfate as a UV absorber able to improve the aging of two cured epoxies. However, increased awareness of the environmental impact of thermosets has raised concerns regarding their use and has pressed the industry and academia to develop tailored recycling strategies for epoxy-based composites. An additional environmental limitation of currently used epoxy systems is the use of petroleum-based raw materials for their synthesis. Life cycle analysis (LCA) must be considered to develop resins and composites complying with the cradle-to-cradle strategy [3].

Rybicka et al. [4] described the technology readiness level (TRL) of several recycling technologies: Incineration and landfilling were classified as TRL 9; pyrolysis for carbon and glass fiber composites resulted in a TRL 8. The fluidized bed pyrolysis and solvolysis process achieved a median TRL of 4. In some recent reviews, the annual capacity of several technologies for recycling carbon-fiber-reinforced composites was discussed [5,6]. Pyrolysis was confirmed as the approach reaching capacities in the range of 1000–2000 tons. The main limitations of thermal and mechanical recycling processes are fibers’ property degradation and that matrices are fully depolymerized with partial recovery in useful forms [7].

Chemical recycling is emerging as a viable approach to recover clean and undamaged reinforcing fibers while allowing for the recovery of monomers or oligomers that can be reused. Xu et al. [8] presented an approach based on the decomposition of epoxy-based composites in a H_2_O_2_/acetone mixed solution heated between 60 and 150 °C. The degraded products were analyzed, showing mainly bisphenol A and its derivatives, such as phenol derivatives, which are generated during the decomposition of the epoxy network. Wang et al. [9] developed a recycling approach based on the use of acetic acid to swell the composites while the weakly coordinating aluminum ions in CH_3_COOH solution selectively cleaved the C−N bond, allowing for obtaining oligomers from the epoxy resins. These papers demonstrated the possibility to cleave epoxy networks, but the reuse strategies for the recovered oligomers were not assessed.

Back in 2012, the company Connora Technologies presented a novel class of ammine reagents named Recyclamine™, designed to be selectively cleaved in an aqueous solution with acetic acid, using mild conditions (i.e., 80 °C), thus yielding clean reinforcing fibers and a reusable thermoplastic matrix from the epoxy network. The Recyclamine™ reactants were characterized in terms of their aging resistance [10], and mixing them with bio-based epoxy monomers, their properties using high-pressure resin transfer molding [11] and resin infusion [12] were measured. The recycling process of bio-based resin cured by Recyclamine™ was investigated by life cycle analysis (LCA), confirming its potential to offer a disruptive solution to the end-of-life problem of epoxy-based composites [10,13,14]. In a recent study, the benefits of Recyclamine™ in terms of life cycle costing (LCC) were assessed [13].

The use of petroleum-based raw materials for the synthesis of epoxy monomers is another limit of the epoxy-based composites used nowadays. To overcome this limit, several researchers developed bio-based epoxy monomers synthetized from vegetable oils [14], natural acids [15,16], lignin [17], and so forth. The procedures for the synthesis of epoxy precursors from natural renewable resources require, in most of the cases, the use of organic solvents limiting the development of a truly green approach. In a recent paper, Esposito Corcione et al. [18] presented an innovative approach to obtain epoxidized monomers starting from waste flours recovered from the processing waste of pasta factories or from the organic fraction of municipal solid waste. This approach simply relies on waste’s treatment with UV/ozone radiations without the use of any solvents. This treatment is fast, cheap, reliable, and with no toxic emissions. The amount of municipal solid waste globally collected per year is approximately 1.3 × 10^12^ t, and it is expected to rise up to 2.2 × 10^12^ t per year by 2025 (EPA (United States Environmental Protection Agency), 2017).

The huge amount of organic waste is becoming an increasing issue for the waste management of modern cities, while the technology developed by Esposito Corcione et al. [18,19] can turn waste into a high-value-added product. However, avoiding global negative impacts when using epoxy monomers is important to develop suitable recycling approaches to reuse epoxy resins at their end of life. This approach respects the cradle-to-cradle strategy.

In the present paper, epoxidized monomers synthetized from waste flour were mixed with bio-based epoxy precursors; then the epoxy blends obtained were cured using a cleavable ammine to develop a fully recyclable bio-based epoxy thermoset. The resins were fully characterized in terms of thermal and mechanical properties to optimize their final performances. The optimized formulation was recycled using only an acidic aqueous solution under mild conditions to demonstrate the possibility to recover a reusable plastic from the cured epoxy resin.

## 2. Materials and Methods

Polar Bear (R-Concept, Barcelona, Spain), a bio-based epoxy system designed specifically for the composite processing.

Recyclamine R-101 (R-Concept, Barcelona, Spain), is a recyclable epoxy cure agent for composite manufacturing. Polar Bear and Recyclamine R-101 are both liquid at room temperature.

Waste flour (WF) was obtained from the processing waste of the pasta factories. Epoxidized waste flour (EWF) was obtained by contemporary exposure to UV radiations and ozone for 5 h following the method reported in a previous work [18] and in a patent application [20]. A medium-pressure Hg UV lamp (UV HG 200 ULTRA, Jelosil Srl, Vimodrone, Italy), with a radiation intensity on the surface of the samples of 9.60 W/mm^2^, was used for waste flour treatment.

FTIR analysis, performed with an FTIR (6300 Spectrometer, Jasco, Cremella, Italy), was used to assess the presence of epoxy groups after UV/ozone exposure. Infrared spectra were recorded in the wavelength range between 400 and 4000 cm^−1^, 128 scans, and 4 cm^−1^ of resolution by using a germanium round crystal window. The spectra acquisition was carried out before and after the UV/ozone and after curing.

As reported in our previous article [20], the epoxy content of the waste flour was checked by using titration, carried out according to Method A of ASTM D 1652-97 (ASTM D (1652)-97, 1997). The amount of the consumed acid during titration, which is an index of the epoxy content of the sample, was used to calculate the epoxy content (E) and the equivalent epoxy weight (WPE) of the waste flour.

Different samples were produced by mixing the Polar Bear resin with specific amounts of epoxidized waste flour and Recyclamine R-101. First, different blends were produced by varying the ratio between Polar Bear and waste flour, keeping a constant amine content (Table 1). The initial amount of amine was chosen by considering the value suggested from R-Concept for the blend of Polar Bear and Recyclamine R-101, corresponding to 22 phr.

After choosing the optimal ratio between Polar Bear and waste flour, the amine content was varied by adding different phr’s of Recyclamine R-101 (Table 2) in order to optimize the curing kinetic of the system.

**Figure 1:**
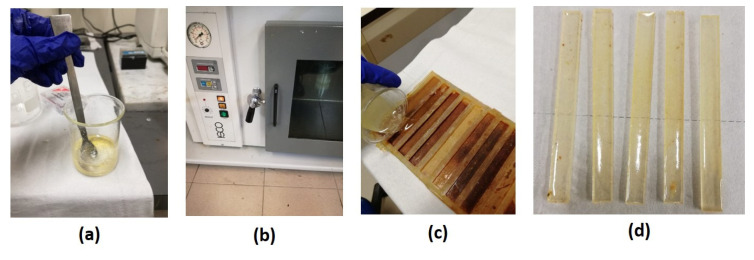
Mixing (**a**), vacuum (**b**), and pouring in silicon molds (**c**) cured thermoset samples (**d**).

All the mixtures were degassed by applying vacuum at room temperature, then pouring in silicon molds and curing for 24 h at room temperature. The cure was followed by a postcure process for 2 h in a static oven. The postcure temperature was varied between 120, 150, and 160 °C, as shown in Figure 1.

### 2.1. Recycling Procedure

Recyclamine^TM^ is an epoxy hardener developed by Connora Technologies that allows for obtaining a recyclable thermoset that can be converted into a meltable thermoplastic. Its recyclability key factor is based on the presence of amino-acid-cleavable groups that allow the cleavage of the crosslink points of the epoxy-cured network [21].

**Figure 2:**
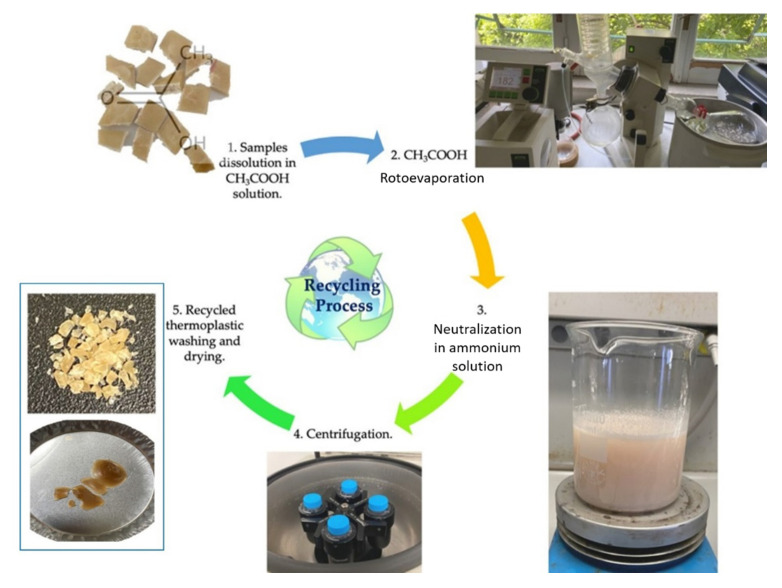
Chemical recycling process’s main steps.

The resin system selected for the recycling trials was EWF50_A15, which showed the best properties among all the resin systems tested, as it will be shown in the paper. The chemical recycling procedure is schematically drawn in Figure 2.

A sample of 5 g of the epoxy system EWF50_A15 was solubilized in 300 mL of 25 %vol acid acetic solution (CH_3_COOH) at 80 °C for 1 h. The obtained mixture was rotoevaporated at 60 °C at a pressure ranging between 110 and 60 mbar and at a rotation speed of 3500 rpm. The distilled acetic acid was stocked, being reusable for a new chemical recycling treatment, while the concentrated solution obtained (about 75 mL) was neutralized in 300 mL of 50 %vol ammonium hydroxide solution. During this phase, a whitish compound started to precipitate, which was the recycled thermoplastic of interest. Then, the solution containing the precipitate was centrifuged for 5 min at 3000 rpm and, at the end, the supernatant removed. The solid phase at the bottom of the test tube was recovered and washed in ionized water to remove any residual traces of acetic acid and ammonium hydroxide solutions. Eventually, it was dried in a vacuum stove for 24 h at 50 °C. The thermoplastic obtained is a brown compact solid shown in the panel of Figure 2. The recycling process applied previously on bio-based epoxy derived from pine oil and paper byproducts [22] resulted in a white solid. However, in this paper we modified the recycling process compared with the one used previously [21] in the following steps: the use of a Rotavapor to concentrate the solution, the replacement of sodium hydroxide with ammonium hydroxide in the neutralization phase, and the use of centrifugation in place of filtration.

The recycling process yield was equal to 85%, in the same range of the yields obtained previously [22]. However, the new process was faster and allowed for reusing the ammonium hydroxide solution, thus leading to a greener process, which is under evaluation using LCA to quantify the environmental benefits.

### 2.2. Methods

Rheological analyses were carried out with a Rheometrics Ares rheometer. A double plate geometry was used, setting a gap of 0.3 mm, constant oscillatory amplitude (1%), and frequency (1 Hz). The tests consisted of a temperature ramp from room temperature to 130 °C.

DSC analysis was performed on a Mettler Toledo 622 differential scanning calorimeter (DSC). Samples were heated from 25 to 250 °C at 20 °C/min in air.

The thermal stability of the films was assessed by TGA, with a TA Instruments SDT Q600 (TA Instruments, New Castle, DE, USA). The samples were heated in an alumina holder from 20 to 600 °C at a heating rate of 10 °C/min under air atmosphere; three measurements were performed on each sample.

The flexural properties of each cured sample were measured using a dynamometer, Lloyd LR5K, according to ASTM D790 (ASTM D790-17, 2017) (three points bending with the specimen dimension: 80 mm × 10 mm × 4 mm). Five replicates were performed on each sample.

Dynamic mechanical analysis was carried out on a dynamic mechanical thermal analyzer (TRITEC2000 by Triton Technology, Leicestershire, UK) by single cantilever geometry. The recycled polymers, after 1 day drying at 40 °C, were tested in their powder form using the pocket DMA approach, a technique used for testing powders in the pharmaceutical field [23] and for polymer blends [24] The polymers obtained from recycling were finely micronized in powder with an average dimension of 30 µm. Then 0.35 g of polymer powder was weighted in a standard stainless steel pocket purchased from Triton and pressed to obtain a uniform thickness. The test was carried out according to the following protocol: the sample was stabilized at 25 °C and then heated up to 180 °C at 5 °C min^−1^; the samples were cooled down naturally and reheated up to 180 °C at 5 °C min^−1^. Similar techniques were also reported by Carlier et al. [25] for organic polymers under the name supported DMA. This kind of technique allows for direct evaluation of thermal transitions from E’ and tan d traces. However, the absolute values of E’ and tan δ for the polymer are influenced by the presence of the metal pocket, and thus, the real values should be analyzed considering the assembly as a sandwich material. The tan δ versus temperature was plotted.

### 2.3. Statistical Analysis

Analysis of variance (ANOVA) was used to highlight the statistical significance of different parameters, as the different amounts of EWF and Polar resin, on the mechanic properties. For this purpose, the F value, which is defined as the ratio of the variation between sample means to the variation within the samples, was calculated from the measured data. Then, being “a” the number of levels of the variance factor and “n” the number of tests for each level, the critical F value, FCV(a-1, a(n-1), α), can be estimated. FCV represents the value of F distribution with degrees of freedom (a-1) and a(n-1), which, at a confidence level, α, corresponds to the null hypothesis (equivalence of the means). Therefore, F < FCV indicates that the population means are equivalent, whereas F > FCV indicates that the population means are significantly different. Another quantitative measure for reporting the result of a test of hypothesis is the *p*-value. The *p*-value is the probability of the test statistic to be at least as extreme as the one observed, given that the null hypothesis is true. A small *p*-value is an indication that the null hypothesis is false. It is good practice to decide in advance of the test how small a *p*-value is required to reject the test, that is, to choose a significance level, α, for the test. For example, it can be decided to reject the null hypothesis if the test statistic exceeds the critical value (for α = 0.05) or, analogously, to reject the null hypothesis if the *p*-value is smaller than 0.05.

## 3. Results and Discussion

**Figure 3:**
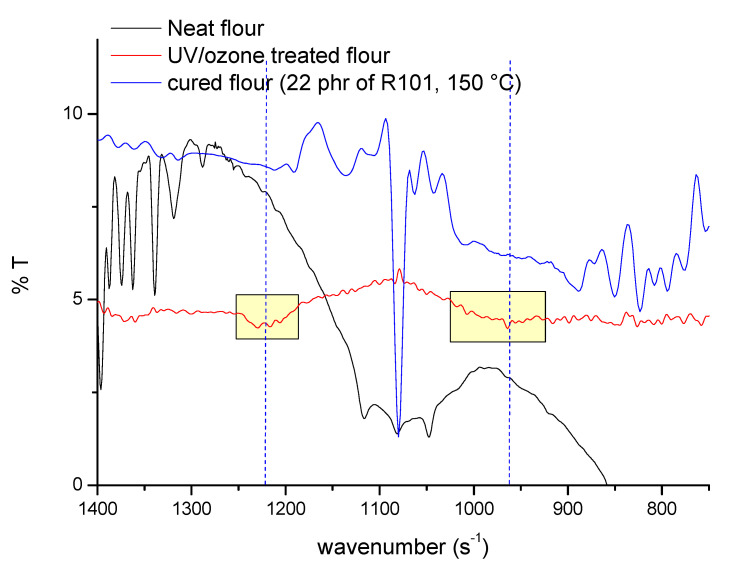
FTIR spectra of waste flour.

The FTIR spectra on waste flour are reported in Figure 3; in particular, the FTIR curve of waste flour (WF) shows the typical peaks of starch: 1412 cm^−1^ assigned to –CH_2_ bending and –COO stretch, 1075, 1048 cm^−1^ and 1022 cm^−1^ assigned to the crystalline and amorphous regions of starch, respectively, and 1164 cm^−1^ assigned to vibrations of the glucosidic C–O–C bond and the whole glucose ring that can present different modes of vibrations and bending conformations.

A contemporary exposure of waste flour to UV/ozone radiation involves the appearance of the typical signals of the epoxy ring at 1260, 890, and 827 cm^−1^. This indicates that the treatment allows for obtaining epoxidized waste flour (EWF).

After curing of the waste flour in the presence of the amine, the peaks due to the epoxy rings disappear, confirming epoxy curing reaction. The strong peak at 1075 cm^−1^ can be again attributed to the bending vibration of residual glucose.

**Figure 4:**
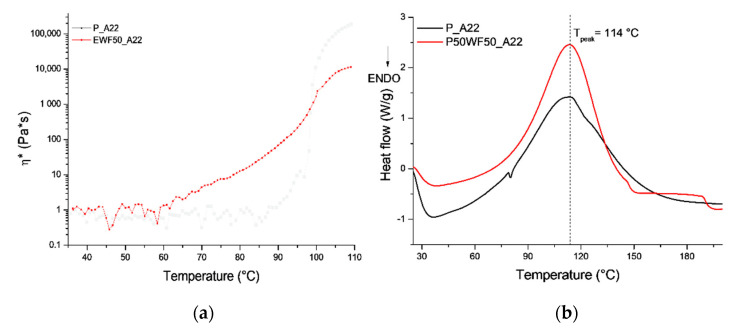
Rheological (**a**) and DSC (**b**) analysis of the curing reaction.

The viscosity curves of the commercial system and its blend with 50% of EWF are reported in Figure 4. The commercial system is characterized by a step increase in viscosity of around 95 °C, which is indicative of the reaction with the amine. The addition of waste flour involves a decrease in the onset temperature of reaction, as clearly observed in Figure 4a. This indicates that in the presence of EWF, the crosslinking reaction of the system is accelerated.

However, as shown in Figure 4b, the exothermal peak temperature obtained by DSC analysis is the same for both systems. A comparison between the rheological and DSC curve of the commercial system shows that viscosity increase occurs around 100 °C, where, however, the extent of reaction, as measured by DSC, is still quite low. This indicates that the rheological analysis can only provide information at a relatively low degree of conversion. The lower onset temperature of viscosity increase observed in Figure 4a for the system with EWF is therefore relative to very low conversions, where probably DSC analysis is not able to detect the very slow heat release of the reaction.

In order to choose the optimal postcure temperature, DSC scans were carried out on systems postcured at three different temperatures (120, 150, and 160 °C) after the cure at room temperature for 24 h.

**Figure 5:**
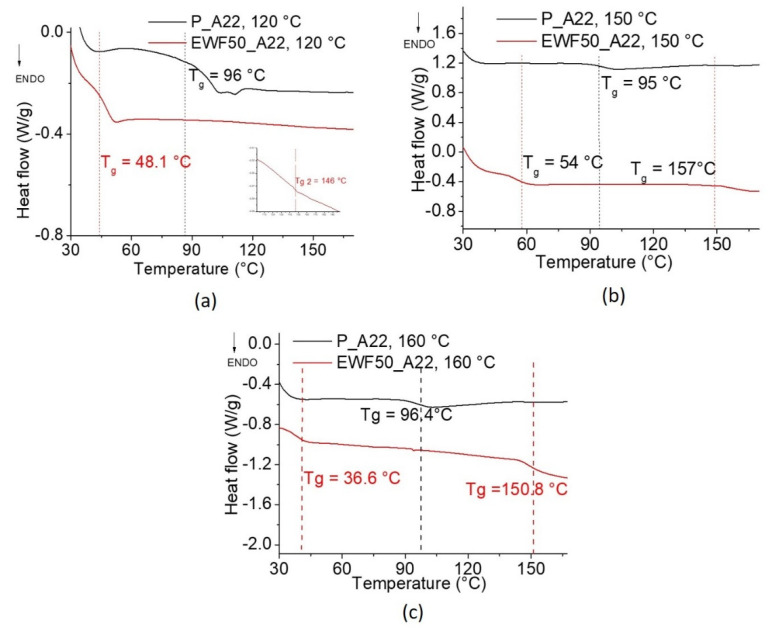
DSC analysis of blends postcured at 120 °C (**a**), 150 °C (**b**), and 160 °C (**c**).

The DSC curves in the temperature range between 30 and 170 °C, reported in Figure 5a–c for the cured systems, show that the postcure temperature has no significant effect on the glass transition of the commercial system, which is, in any case, around 96 °C. On the other hand, two different glass transition signals were detected in the blend with EWF, which indicates a partial miscibility of the system. Both Tg values were significantly affected by the postcure temperature. The higher values were found after postcure at 150 °C. The decrease of the Tg values after further increasing the postcure at 160 °C was due to the poor thermal stability of the EWF, which, from TGA analysis, was found to have an onset temperature of degradation of around 170 °C.

With the aim of developing an epoxy system that could find use in different industrial applications, a glass transition at around 140–150 °C is high enough to guarantee good performances and stability of properties. On the other hand, the lower Tg represents a limit of the developed system.

Therefore, our further efforts were aimed at increasing the lower Tg signal. In the following analysis, we will only focus on the lower Tg, neglecting potential changes of the higher Tg.

**Figure 6:**
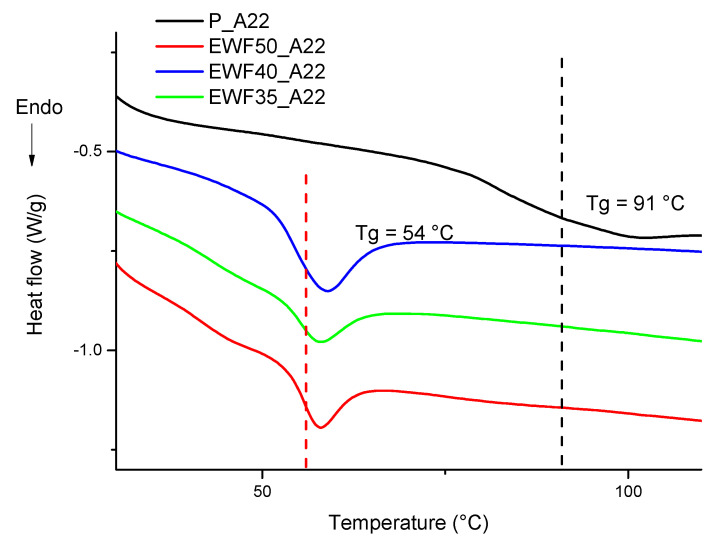
DSC analysis of postcured blends at different EWF contents.

Figure 6 shows DSC heating scans on samples postcured at 150 °C with different amounts of waste flour. No difference in the lower glass transition temperature was detected with increasing EWF content. Therefore, in order to increase the amount of recycled material, we focused our further analysis on the blend at 50% of EWF. Unfortunately, it was impossible to further increase the amount of EWF since this resulted in a significant increase in the liquid blend viscosity. However, the Tg value of the blends was much lower than that of the neat commercial systems, which required further optimization of the amine content in order to increase the glass transition of the system.

**Figure 7:**
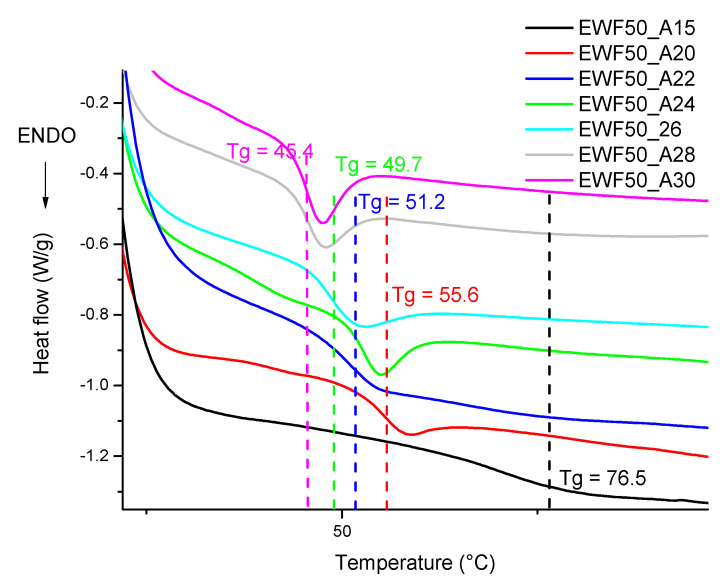
DSC analysis of postcured blends at different amine contents.

**Figure 8:**
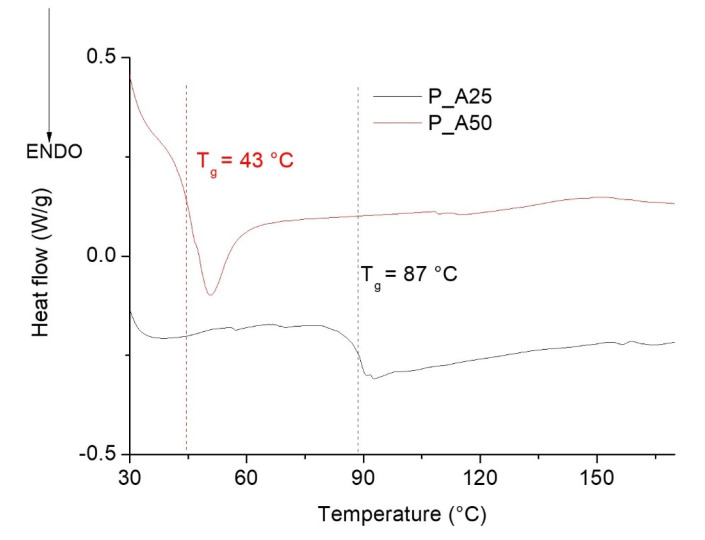
DSC analysis of postcured commercial epoxy at different amine contents.

In Figure 7, the DSC curves of samples at a constant EWF content and varying amine amounts are reported. A lower amine amount allowed for increasing the lower glass transition of the system. This indicates that when the amount of amine was too high, an excess of uncured amine remained in the sample after curing. This amine can effectively act as a plasticizer for the epoxy, significantly reducing the glass transition signal. A similar behavior was in fact observed for the commercial system at higher amine contents, as reported in Figure 8. Additionally, in this case, the excess of unreacted amine caused a plasticization effect, which reduced the Tg of the system. According to our analysis, it would be possible to further reduce the amount of amine. However, for the same reason previously discussed, the amount of amine was not further reduced below 15 phr because it resulted in very high viscosities.

**Figure 9:**
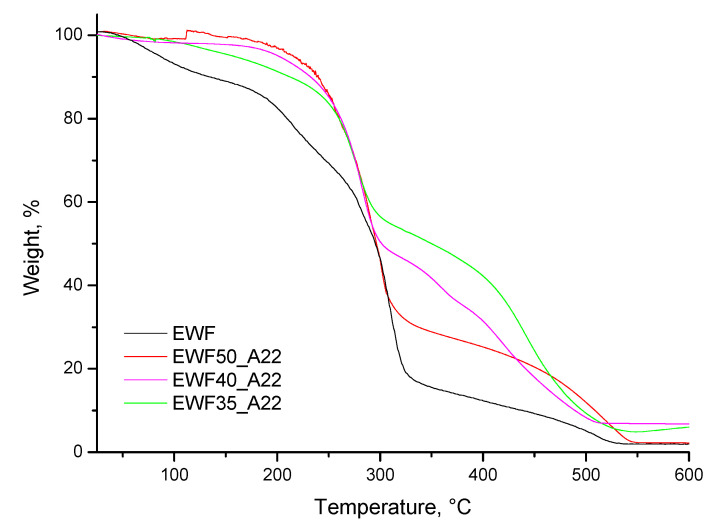
TGA on EWF with different Polar resin contents.

Thermogravimetric analyses, shown in Figure 9, were performed on EWF without and with the addition of different amounts of Polar resin. The EWF sample showed a first weight loss below 150 °C, mainly attributed to the water evaporation, and a second loss between 150 and 350 °C, due to the degradation of the starch. The addition of Polar resin involved, in any case, a strong decrease in the absorption of water. Additionally, an increase in thermal stability was detected in the second stage, between 250 and 350 °C, where the production of a carbonaceous residue at lower temperatures occurred together with the degradation of the flour. In this step, a decrease in weight loss was detected by increasing the Polar resin content. The second stage was followed by a third degradation step, characterized by the oxidation of the remaining carbonaceous char; also in this step, the production of a higher final solid residue was detected with higher Polar content.

**Figure 10:**
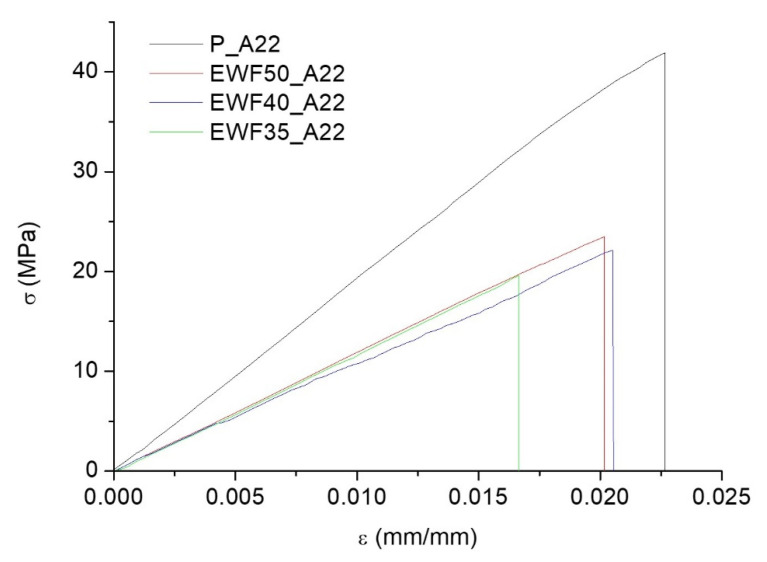
Stress–strain curves from flexural tests of cured blends at different EWF contents.

In Figure 10, the stress–strain curves obtained from flexural tests on samples with different EWF contents are reported. Results from flexural tests, and the mechanical properties reported in Table 3, confirmed the results from DSC analysis. The lower Tg found in Figure 6 for the blends, compared with the neat commercial system, resulted in lower mechanical properties too. The flexural modulus and strength of the blends were significantly lower compared with the commercial system. This was first due to the structure of the samples. In particular, the structure of the manufactured system was influenced by the presence of a crystalline zone of the waste flour typical of starch, which remained unchanged even after the progressive reprocessing cycles. As reported in our previous work [20], waste flour showed a semicrystalline nature, without any significant change in the crystalline fraction and the crystal planes, compared with native starch. The presence of this crystalline fraction involved an increase in brittleness of the sample, compared with the completely amorphous Polar Bear–Recyclamine system. On the other hand, the addition of Polar Bear resin involved a strong increase in the mechanical response, compared with the results found in our previous work [20] for the sample made up of only waste flour, characterized by very low flexural strength (7.32 ± 0.65 MPa). The addition of Polar resin, in fact, allowed a reduction of the high number of voids and defects occurring during both the water evaporation and the curing process of neat waste flour samples.

This was confirmed by one-way analysis of variance (ANOVA). Considering the amount of EWF as the source of variation, with four levels, and three degrees of freedom, its significance on flexural modulus, strength, and strain at break was tested by calculating the F value as the ratio of the variance between the means to the variance of the experimental error. The F value was then used in order to calculate the corresponding *p*-value, which was then compared with the confidence level, α = 0.05. According to ANOVA, *p* > α corresponds to the null hypothesis (equivalence of the means), whereas *p* < α indicates that the population means were significantly different. For flexural strength and modulus, *p* = 0.0013 and *p* = 0.01, respectively, indicate the statistically relevant effect of the addition of EWF on the corresponding property of the blend. In contrast, for strain at break, *p* = 0.39 indicates that the effect of EWF was not statistically significant. 

On the other hand, limiting the ANOVA to the blends, and therefore neglecting the sample of commercial epoxy, with three levels, and two degrees of freedom, for flexural strength and modulus, *p* = 0.85 and *p* = 0.50, respectively, indicates that the corresponding mechanical properties were not influenced by the amount of EWF in the tested range of compositions. This is consistent with the fact that, in Figure 6, the lower Tg signal was independent on the amount of EWF.

**Figure 11:**
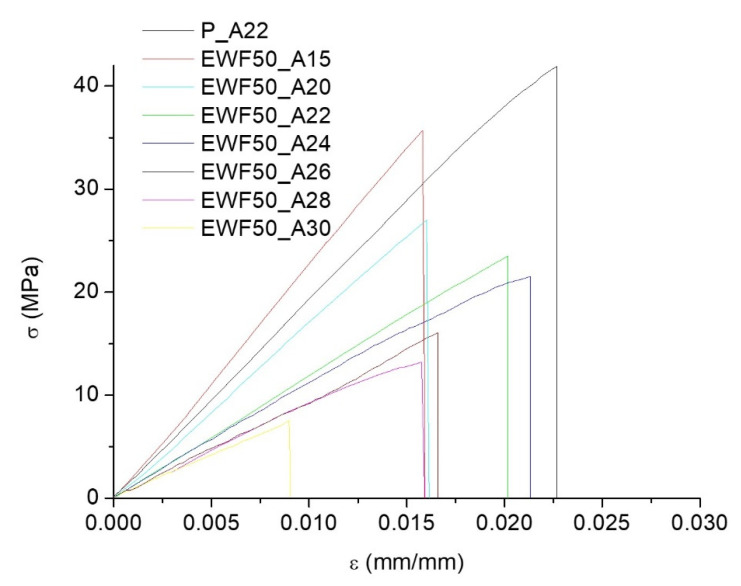
Stress–strain curves from flexural tests of cured blends at different amine contents.

In Figure 11, samples with different amine contents are compared. The mechanical properties calculated from the stress–strain curves are reported in Table 4. ANOVA was again used to establish the effect of the amount of amine on the mechanical properties of the sample. Considering the data in Table 4, neglecting the commercial sample, and therefore considering seven levels, and six degrees of freedom, for flexural strength, modulus, and strain at break, *p* = 2.1 × 10^−11^, *p* = 8.3 × 10^−6^, and *p* = 8.2 × 10^−4^, respectively, indicate that the amount of amine had a statistically significant effect on each of the mechanical properties. Referring to the data in Table 4, each of the mechanical properties increased with a decreasing amount of amine. The lower modulus and strength found for the higher amine content confirmed that excess amine acts as a plasticizer for the cured epoxy, which, as discussed for Figure 7, involved the Tg reduction.

In addition, ANOVA was used to compare only the commercial system with the blend at 15 phr of ammine. With two levels, and one degree of freedom, for flexural strength, modulus, and strain at break, *p* = 0.43, *p* = 0.8, and *p* = 0.61 highlight that the two samples were not statistically different. This is consistent with the observation from DSC analysis, which showed that, for the sample at 15 phr amine, a relatively high Tg was found, which was only 15 °C lower than that of the commercial system. All the ANOVA findings are summarized in Table 5, Table 6, Table 7 and Table 8.

**Figure 12:**
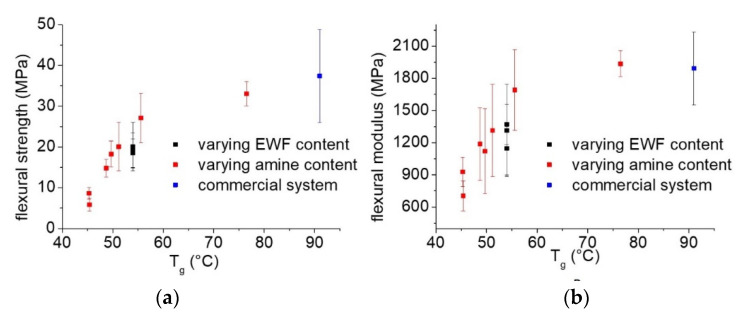
Effect of the lower-range Tg on the evolution of (**a**) flexural strength and (**b**) flexural modulus.

The effect of the lower Tg of the blends on the flexural strength and modulus is also highlighted in Figure 12a,b. From the plots, it is clear that all the samples produced at different EWF or amine amounts fall on a single master curve. Interestingly, also the commercial epoxy falls on the same master curve. This indicates that the low-range Tg is the parameter that mainly influenced the mechanical properties of the produced resin. The higher-range Tg (measured around 150 °C) and the chemical structure of the resulting polymer had only marginal effects on the flexural strength and modulus of the produced blends.

**Figure 13:**
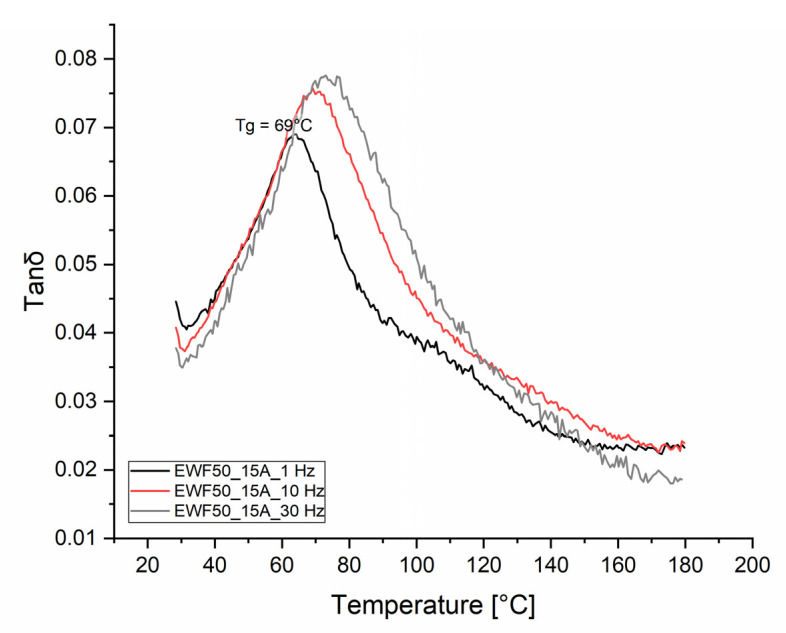
Tan δ vs. temperature of the polymer obtained from the recycling of the EWF50_A15 formulation.

The recycled polymer obtained from the chemical recycling of the epoxy network was characterized by DMA and DSC. The tan δ vs. temperature was measured at three different frequencies: 1, 10, and 30 Hz. The results (Figure 13) clearly showed the presence of a single peak for all the frequencies tested. The peak was centered at 69 °C at 1 Hz, and it shifted to higher temperatures for increasing frequencies. This relaxation behavior was shown by glass transition temperature (Tg). Similar values were typically displayed by similar polymers derived from the recycling of bio-based epoxies cured by cleavable ammines [12]. Reprocessable bio-based epoxy cured using an aromatic disulfide crosslinker with diacid functionality displayed, after recycling, a glass transition temperature between 65 and 73 °C [26]. Slightly higher (i.e., 80 °C) and even lower (i.e., 18 °C) Tg values were obtained by the same group, varying the epoxy precursor among different epoxidized linseed and soybean oils cured by 2,2′-dithiodibenzoic acid [27].

## 4. Conclusions

An exemplary bio-based epoxy resin formulation showing full recyclability was presented in this paper. Two different epoxy resin precursors were mixed with a cleavable ammine: a commercial bio-based epoxy and a novel epoxidized waste flour. The latter chemical reactant was obtained using a green approach based on the use of a UV/ozone treatment. Mixing the two epoxy monomers allowed for obtaining formulations easy to mix at room temperature showing low viscosity in the unreacted state that could be cured at temperatures varying between 120, 150, and 160 °C. 

The glass transition temperature of the epoxy formulation was optimized by varying the ratio between the two epoxy monomers and the amount of the cleavable amine. When the ammine was added at 22 phr, the glass transition temperature was fixed at about 54 °C with no significant change varying the bio-based epoxy content. However, reducing the ammine content down to 15 phr improved the glass transition temperature up to 76.5 °C, while increasing the ammine content to 30 phr reduced the Tg down to 45.4 °C. Similar results were obtained when considering the mechanical properties with the formulation cured with 15 phr ammine content. This hardener amount was chosen as optimal since a further reduction of the ammine content resulted in high viscosity systems.

The resin developed showed optimal recyclability when treated with an acidic solution under mild conditions (i.e., 80 °C for 1 h), obtaining a plastic material with a Tg of 69 °C by DMA and about 50 °C by DSC. The plastic material obtained from the recycling is unique compared with those obtained before as it was derived from a valuable thermoset formulated using 50 wt% of an epoxy monomer synthetized from waste flours.

The use of materials derived from organic waste as valuable thermoset prepolymer, which, after curing, can also be recycled at the end of their life, can truly revolutionize the recycling strategies for organic wastes, avoiding negative impacts on the environment. At the same time, the possibility to recover a high percentage (i.e., 85%) of this product into a reusable plastic matrix can guarantee the respect of the environment. However, more efforts will be required to further optimize the thermal and mechanical properties of the cured thermosets. These efforts should be focused on the synthesis of reactants, leading to a balanced stoichiometry and with a stiffer structure to improve the glass transition temperature and the mechanical properties.

## 5. Patents

Esposito Corcione, C., Greco, A., Visconti, P., Striani, R., Ferrari, F., 2019. Process for the production of bio-resins and bio-resins thus obtained. 102019000016151. IT.

## Figures and Tables

**Table 1:** Samples at different commercial/epoxidized waste flour ratios and constant amine content.

Sample Name	Polar Bear (wt%)	Epoxidized Waste Flour (wt%)	Recyclamine R-101 (phr)
P_A22	100	-	22
EWF35_A22	65	35	22
EWF40_A22	60	40	22
EWF50_A22	50	50	22

**Table 2:** Samples at constant commercial/epoxidized waste flour ratio and varying amine contents.

Sample Name	Polar Bear (%)	Epoxidized Waste Flourn (%)	Recyclamine R-101 (phr)
EWF50_A15	50	50	15
EWF50_A20	50	50	20
EWF50_A22	50	50	22
EWF50_A24	50	50	24
EWF50_A26	50	50	26
EWF50_A28	50	50	28
EWF50_A30	50	50	30

**Table 3:** Flexural properties of cured blends at different EWF contents.

Sample	σ_R_ (MPa)	ε_R_ (mm/mm)	E (MPa)
P_A22	37.4 ± 11.4	0.021 ± 0.008	1893 ± 340
EWF50_A22	20.1 ± 5.9	0.020 ± 0.004	1313 ± 430
EWF40_A22	18.5 ± 3.5	0.021 ± 0.007	1146 ± 245
EWF35_A22	19.1 ± 4.3	0.014 ± 0.009	1371 ± 188

**Table 4:** Flexural properties of cured blends at different amine contents.

Sample	σ_R_ (MPa)	ε_R_ (mm/mm)	E (MPa)
P_A22	37.4 ± 11.4	0.021 ± 0.008	1893 ± 340
EWF50_A 15	33.0 ± 2.9	0.019 ± 0.003	1935 ± 120
EWF50_A 20	27.1 ± 6.0	0.021 ± 0.003	1690 ± 377
EWF50_A 22	20.1 ± 5.9	0.020 ± 0.004	1313 ± 430
EWF50_A 24	18.3 ± 3.2	0.018 ± 0.001	1121 ± 397
EWF50_A 26	14.8 ± 2.2	0.016 ± 0.008	1188 ± 339
EWF50_A 28	8.60 ± 1.4	0.012 ± 0.003	927 ± 136
EWF50_A 30	5.84 ± 1.5	0.010 ± 0.002	705 ± 140

**Table 5:** ANOVA parameters—amount of EWF as the source of variation.

Property	Source of Variation	SS	MS	*F*-Test	*p*-Value
Flexural strength	EWF amount (4 levels)	1244	414	8.48	0.0013
	Error	782	48.8		
Flexural modulus	EWF amount (4 levels)	1.56 × 10^6^	5.2 × 10^5^	5.25	0.010
	Error	1.58 × 10^6^	9.89 × 10^4^		
Strain at break	EWF amount (4 levels)	0.00017	5.7 × 10^−5^	1.08	0.385
	Error	0.00084	5.3 × 10^−5^		

**Table 6:** ANOVA parameters—amount of EWF as the source of variation, neglecting the sample of commercial epoxy.

Property	Source of Variation	SS	MS	*F*-Test	*p*-Value
Flexural strength	EWF amount (3 levels)	6.53	3.26	0.149	0.862
	Error	262	21.85		
Flexural modulus	EWF amount (3 levels)	1.36 × 10^5^	6.82 × 10^4^	0.73	0.502
	Error	1.12 × 10^6^	9.34 × 10^4^		
Strain at break	EWF amount (3 levels)	3.3 × 10^−6^	1.7 × 10^−6^	0.0387	0.962
	Error	0.00052	4.3 × 10^−5^		

**Table 7:** ANOVA parameters—effect of the amount of amine on the mechanical properties.

Property	Source of Variation	SS	MS	*F*-Test	*p*-Value
Flexural strength	Amine amount (8 levels)	2791	465	33.1	1.84 × 10^−11^
	Error	394	14		
Flexural modulus	Amine amount (8 levels)	5.43 × 10^6^	9.05 × 10^5^	9.71	8.35 × 10^−6^
	Error	2.61 × 10^6^	9.31 × 10^4^		
Strain at break	Amine amount (8 levels)	5.24 × 10^−4^	8.6 × 10^−5^	5.40	8.18 × 10^−4^
	Error	4.56 × 10^−4^	1.6 × 10^−5^		

**Table 8:** ANOVA parameters—effect of the amount of amine on the mechanical properties, neglecting samples with commercial epoxy.

Property	Source of Variation	SS	MS	*F*-Test	*p*-Value
Flexural strength	Amine amount (2 levels)	48.4	48.4	0.699	0.427
	Error	553	69.2		
Flexural modulus	Amine amount (2 levels)	4410	4410	0.0678	0.801
	Error	5.20 × 10^5^	6.5 × 10^4^		
Strain at break	Amine amount (2 levels)	0.00001	0.00001	0.274	0.615
	Error	0.00029	3.7 × 10^−5^		

## Data Availability

Not applicable.

## References

[1] Zotti A., Zuppolini S., Borriello A., Zarrelli M. (2020). Thermal and mechanical characterization of an aeronautical graded epoxy resin loaded with hybrid nanoparticles. Nanomaterials.

[2] Awad S.A., Mahini S.S., Fellows C.M. (2019). Modification of the resistance of two epoxy resins to accelerated weathering using calcium sulfate as a photostabilizer. J. Macromol. Sci. Part A.

[3] La Rosa A.D., Cicala G., Muthu S.S. (2015). Handbook of Life Cycle Assessment (LCA) of Textiles and Clothing.

[4] Rybicka J., Tiwari A., Leeke G.A. (2016). Technology readiness level assessment of composites recycling technologies. J. Clean. Prod..

[5] Giorgini L., Benelli T., Brancolini G., Mazzocchetti L. (2020). Recycling of carbon fiber reinforced composites waste to close their Life Cycle in a Cradle-to-Cradle approach. Curr. Opin. Green Sustain. Chem..

[6] Zhang J., Chevali V.S., Wang H., Wang C.H. (2020). Current status of carbon fibre and carbon fibre composites recycling. Compos. Part B Eng..

[7] Pimenta S., Pinho S.T. (2011). Recycling carbon fibre reinforced polymers for structural applications: Technology review and market outlook. Waste Manag..

[8] Li J., Xu P.-L., Zhu Y.-K., Ding J.-P., Xue L.-X., Wang Y.-Z. (2012). A promising strategy for chemical recycling of carbon fiber/thermoset composites: Self-accelerating decomposition in a mild oxidative system. Green Chem..

[9] Wang Y., Cui X., Ge H., Yang Y., Wang Y., Zhang C., Li J., Deng T., Qin Z., Hou X. (2015). Chemical Recycling of Carbon Fiber Reinforced Epoxy Resin Composites via Selective Cleavage of the Carbon–Nitrogen Bond. ACS Sustain. Chem. Eng..

[10] Cicala G., La Rosa A.D., Latteri A., Banatao R., Pastine S. The use of recyclable epoxy and hybrid lay up for biocomposites: Technical and LCA evaluation. Proceedings of the CAMX 2016—Composites and Advanced Materials Expo.

[11] Cicala G., Mannino S., La Rosa A.D., Banatao D.R., Pastine S.J., Kosinski S.T., Scarpa F. (2017). Hybrid biobased recyclable epoxy composites for mass production. Polym. Compos..

[12] Cicala G., Pergolizzi E., Piscopo F., Carbone D., Recca G. (2018). Hybrid composites manufactured by resin infusion with a fully recyclable bioepoxy resin. Compos. Part B Eng..

[13] Daniela A., Rosa L., Greco S., Tosto C., Cicala G. (2021). LCA and LCC of a chemical recycling process of waste CF-thermoset composites for the production of novel CF-thermoplastic composites. Open loop and closed loop scenarios. J. Clean. Prod..

[14] Tan S.G., Chow W.S. (2010). Biobased epoxidized vegetable oils and its greener epoxy blends: A review. Polym.-Plast. Technol. Eng..

[15] Ma S., Liu X., Jiang Y., Tang Z., Zhang C., Zhu J. (2013). Bio-based epoxy resin from itaconic acid and its thermosets cured with anhydride and comonomers. Green Chem..

[16] Aouf C., Nouailhas H., Fache M., Caillol S., Boutevin B., Fulcrand H. (2013). Multi-functionalization of gallic acid. Synthesis of a novel bio-based epoxy resin. Eur. Polym. J..

[17] Llevot A., Grau E., Carlotti S., Grelier S., Cramail H. (2016). From Lignin-derived Aromatic Compounds to Novel Biobased Polymers. Macromol. Rapid Commun..

[18] Esposito Corcione C., Ferrari F., Striani R., Visconti P., Greco A. (2020). Recycling of organic fraction of municipal solid waste as an innovative precursor for the production of bio-based epoxy monomers. Waste Manag..

[19] Ferrari F., Striani R., Minosi S., de Fazio R., Visconti P., Patrono L., Catarinucci L., Esposito Corcione C., Greco A. (2020). An innovative IoT-oriented prototype platform for the management and valorization of the organic fraction of municipal solid waste. J. Clean. Prod..

[20] Esposito Corcione C., Greco A., Visconti P., Striani R., Ferrari F. (2019). Process for the Production of Bio-Resins and Bio-Resins thus Obtained. IT.

[21] La Rosa A.D., Banatao D.R., Pastine S.J., Latteri A., Cicala G. (2016). Recycling treatment of carbon fibre/epoxy composites: Materials recovery and characterization and environmental impacts through life cycle assessment. Compos. Part B Eng..

[22] Pastine S.J. (2018). Sterically Hindered Aliphatic Polyamine Cross-Linking Agents, Compositions Containing and Uses Thereof. U.S. Patent.

[23] Mahlin D., Wood J., Hawkins N., Mahey J., Royall P.G. (2009). A novel powder sample holder for the determination of glass transition temperatures by DMA. Int. J. Pharm..

[24] Cicala G., Mamo A., Recca G., Restuccia C.L. (2007). Synthesis and thermal characterization of some novel ABA block copolymers. Macromol. Mater. Eng..

[25] Carlier V., Sclavons M., Legras R. (2001). Supported dynamic mechanical thermal analysis: An easy, powerful and very sensitive technique to assess thermal properties of polymer, coating and even nanocoating. Polymer (Guildf.).

[26] Di Mauro C., Genua A., Rymarczyk M., Dobbels C., Malburet S., Graillot A., Mija A. (2021). Chemical and mechanical reprocessed resins and bio-composites based on five epoxidized vegetable oils thermosets reinforced with flax fibers or PLA woven. Compos. Sci. Technol..

[27] Di Mauro C., Malburet S., Graillot A., Mija A. (2020). Recyclable, Repairable, and Reshapable (3R) Thermoset Materials with Shape Memory Properties from Bio-Based Epoxidized Vegetable Oils. ACS Appl. Bio Mater..

